# Test utilization for the diagnosis of vitamin B12 and folate deficiency in local clinics in Korea

**DOI:** 10.1002/jcla.23441

**Published:** 2020-11-06

**Authors:** Rihwa Choi, Youngju Oh, Mi‐Jung Park, Serim Kim, Youngrae Kim, Sang Gon Lee, Eun Hee Lee

**Affiliations:** ^1^ Department of Laboratory Medicine Green Cross Laboratories Yongin Korea; ^2^ Department of Laboratory Medicine and Genetics Samsung Medical Center Sungkyunkwan University School of Medicine Seoul Korea; ^3^ Green Cross Laboratories Yongin Korea

**Keywords:** folate, homocysteine, methylmalonic acid, test utilization, vitamin B12

## Abstract

**Background:**

Current guidelines pertaining to diagnosing macrocytic anemia in association with vitamin B12 and folate deficiency recommend that vitamin B12, folate, homocysteine, and methylmalonic acid assays should be assessed concurrently due to their close relationship in metabolism. We aimed to investigate the completion of these assays in local clinics and hospitals without in‐house clinical laboratories in Korea.

**Methods:**

We retrospectively reviewed data from the laboratory information system between September 25, 2017, and June 30, 2019, to investigate usage rates of vitamin B12, folate, homocysteine, and methylmalonic acid assays in patients with macrocytic anemia.

**Results:**

During the study period, 14 894 Korean adults among 109 524 (13.6%) total hemoglobin‐tested subjects underwent concurrent erythrocyte mean corpuscular volume (MCV) tests. Among these 14,894 adults, 265 (1.2%) from 94 local clinics or hospitals without in‐house clinical laboratories in Korea had macrocytic anemia. Furthermore, among these 265 adults, only one woman underwent serum vitamin B12 and folate assay and one man underwent serum homocysteine testing during the study period. No patients among the 265 individuals with macrocytic anemia received erythrocyte folate or methylmalonic acid testing (with either serum, plasma, random urine, or 24‐hour collected urine).

**Conclusions:**

The results of this study provide basic information regarding utilization rates of assays in association with vitamin B12 and folate deficiency. Making more data available is expected to improve rates of testing in patients with macrocytic anemia in local clinics and hospitals without in‐house clinical laboratories in Korea.

## 1 INTRODUCTION

Folate is essential for the prevention of a wide spectrum of health issues, including, most notably, megaloblastic anemia and neural tube defects.^1^ Neural tube defects are major birth defects of the brain and spine that occur early in pregnancy due to improper closure of the embryonic neural tube, which may lead to a range of disabilities or death of the infant.^2^ Vitamin B12 is a cofactor of methionine synthase and L‐methylmalonyl–coenzyme A mutase, and the interaction between folate and vitamin B12 is responsible for the megaloblastic anemia seen in both vitamin deficiencies.^3^ Vitamin B12 is also necessary for the development and initial myelination of the central nervous system as well as for the maintenance of its normal function; thus, vitamin B12 deficiency has been known to causes megaloblastic anemia, demyelinating neurologic disease, or both.^3^ Current guidelines for diagnosing vitamin B12 and folate deficiency recommend that vitamin B12 and folate assays should be conducted concurrently due to the close relationship in metabolism.^4^ However, although often used as the first‐line screening test for vitamin B12 or folate deficiency, serum vitamin B12 or folate in serum and/or in erythrocyte measurement used in isolation show generally poor sensitivities and specificities for the reliable detection of vitamin B12 or folate deficiency.^5^ Since 5‐methyltetrahydrofolate, the main form of folate found in blood, is essential for the vitamin B12‐dependent methionine synthase‐mediated remethylation of homocysteine to methionine, the measurement of methylmalonic acid, homocysteine, or both is used to confirm vitamin B12 deficiency in untreated patients; thus, an elevated level of methylmalonic acid is more sensitive and specific for the diagnosis.^1^, ^3^

Test utilization necessitates not only reducing costs by eliminating unnecessary testing but also selecting the most appropriate tests to drive value and improve patient outcomes.^6^, ^7^ Test “underutilization” occurs when a provider does not request a test that could positively affect patient outcomes, which may lead to an incorrect or delayed diagnosis.^6^, ^8^ To improve the quality of clinical laboratories, improving the assessment and understanding of the current status of testing are essential for establishing meaningful metrics and monitoring the effectiveness of a particular action implemented to alter said rates of utilization.^6^, ^9^ However, to the best of our knowledge, only limited data suggesting the conduct of vitamin B12, folate, homocysteine, and methylmalonic acid assays to diagnose and manage patients with macrocytic anemia in association with vitamin B12 and folate deficiency in Korean adults exist at this time.

Thus, in this study, we investigated the completion rates of vitamin B12, folate, homocysteine, and methylmalonic acid assays for diagnosing and managing Korean adult patients with macrocytic anemia in association with vitamin B12 and folate deficiency who visited local clinics and hospitals without their own clinical laboratories for the first time in Korea.

## 2 MATERIALS AND METHODS

We retrospectively reviewed the test results from Korean adults who underwent hemoglobin (Hb) testing between September 25, 2017, and June 30, 2019, through the laboratory information system of Green Cross Laboratories. Green Cross Laboratories, one of the largest referral clinical laboratories in South Korea, provides clinical specimen analysis services including serum folate, erythrocyte folate, serum homocysteine, and methylmalonic acid (ie, serum, plasma, random urine, and 24‐hour collected urine) tests; complete blood count findings including Hb; and erythrocyte mean corpuscular volume (MCV) to clinics and hospitals nationwide. Missing data for age or sex were excluded. All data were anonymized before being adopted for statistical analysis. The protocol of this study was approved by the institutional review board (IRB) of Green Cross Laboratories (IRB GCL‐2020‐1014‐01). A waiver of informed consent was approved by the IRB since the use of a waiver would not adversely affect the rights or welfare of the study subjects because the study was retrospective and involved no more than minimal risk to the subjects. The study was conducted in accordance with the Declaration of Helsinki. Hb and MCV were analyzed using Sysmex XN 9000 analyzers. The reference intervals of Hb and MCV for adults were as follows: 11.0 to 15.0 g/dL of Hb in women, 13.0 to 17.0 g/dL of Hb in men, and 80 to 102 fL for MCV in all adults. Serum and erythrocyte folate were determined using the Elecsys Folate assay traceable to World Health Organization (WHO) International Standard (IS) 03/178 on Cobas 8000 e801 analyzers (Roche Diagnostics, Germany). Serum homocysteine level was determined using the AutoLab Homocysteine assay (IVD‐LAB, South Korea) on Cobas 8000 c702 analyzers (Roche Diagnostics, Germany) traceable to standard reference material 1955. Methylmalonic acid concentration in serum, plasma, random urine, and 24‐hour collected urine samples was measured using gas chromatography‐mass spectrometry analyzers (Clarus 680; PerkinElmer, USA) with isotope‐labeled internal standards, which were developed and validated by Green Cross Laboratories (detailed information of the method available upon reasonable request to the corresponding authors). The accuracy of the assays during the study period was maintained using the Proficiency Testing/Quality Management program of the College of American Pathologists survey in the United States. Age among the overall subjects was not normally distributed. The numbers and percentages of each test considered were presented in this study. Mann‐Whitney U test when appropriate was adopted to compare age in sex groups. All *P*‐values of less than 0.05 were considered to be significant. Statistical analyses were executed using Microsoft Excel 2019 and MedCalc version 19.1.5 (MedCalc Software bv, Ostend, Belgium).

## 3 RESULTS

**FIGURE 1:**
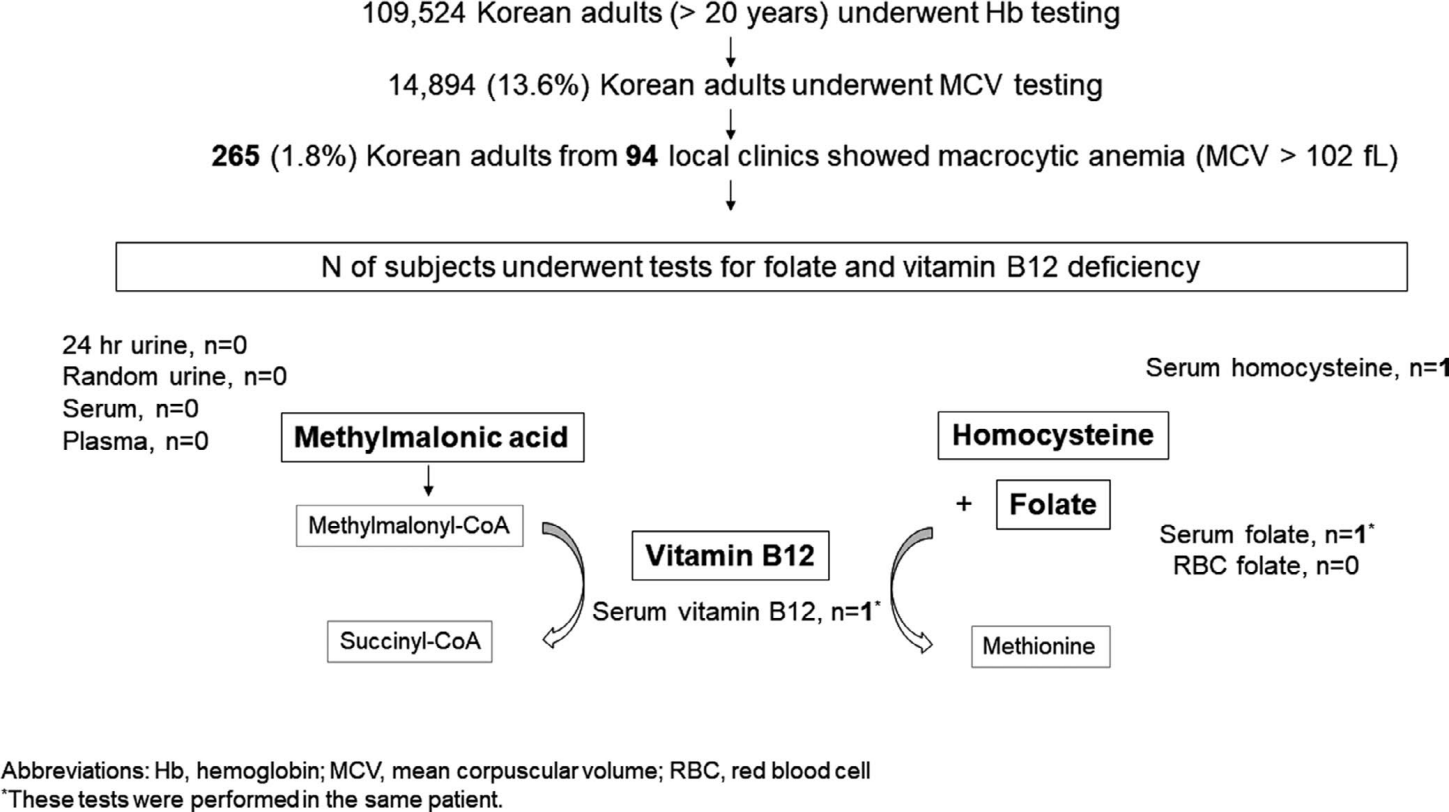
Schematic diagram of this study

During the study period, 109,524 Korean adults completed Hb tests. Among them, 14 894 (13.6%) received concurrent erythrocyte MCV tests. Among these 14 894 adults, 265 adults (107 men and 158 women; 1.2%) aged 23.9 to 95.4 years (median: 68.8 years) had macrocytic anemia, showing Hb findings below the lower limit of the reference interval for each sex and gender and MCV results of greater than 102 fL, from among 94 local clinics or hospitals without in‐house clinical laboratories in Korea. Age was not significantly different among the patients with macrocytic anemia (*P* > .05). Among 265 patients with macrocytic anemia, only one woman underwent both serum vitamin B12 (464 pg/mL, reference interval: 197‐771 pg/mL) and folate (3 ng/mL, reference interval: 3.9‐26.8 ng/mL) assays. Separately, one man from a different clinic underwent serum homocysteine testing during the study period (12.0 µmol/L; upper reference limit: 15.4 µmol/L) and his results were within the reference interval. No patients among 265 patients with macrocytic anemia underwent folate in erythrocytes or methylmalonic acid testing (with either serum, plasma, random urine, or 24‐hour collected urine; Figure 1).

## 4 DISCUSSION

In this study, we investigated the adoption of vitamin B12, folate, homocysteine, and methylmalonic acid assays in association with vitamin B12 and folate deficiency in adult patients with macrocytic anemia in local clinics and hospitals without in‐house clinical laboratories in South Korea. To the best of our knowledge, this is the first study to focus on the rates of vitamin B12, folate, homocysteine, and methylmalonic acid assays performed in patients with macrocytic anemia visiting local clinics and hospitals.

In this study, the adoption of serum vitamin B12 and folate and serum homocysteine assays was below 0.5% among all patients with macrocytic anemia (1/295) and 1.1% (1/94 clinic each) for total clinics and hospitals while those rates of erythrocyte folate and methylmalonic acid tests in varied specimen types were 0.0%. During the study period, methylmalonic acid assays using varied specimen types were conducted by university and hospitals with own clinical laboratories. Although the scope of this study was to examine test utilization in local clinics without their own clinical laboratories, during the study period, 259 patients were tested for methylmalonic acid using 24‐hour urine and 13 of them presented elevated results (>9 mg/d; 5.0%), 2,361 patients were tested for methylmalonic acid by random urine and 472 of them had elevated results (>3.76 mg/g creatinine; 20.0%), 144 patients were tested for methylmalonic acid in plasma and 20 of them had elevated results (>0.4 µmol/L; 13.9%), and 151 patients were tested for methylmalonic acid in serum and 28 had elevated results (>0.4 µmol/L; 18.5%) from university hospitals and other hospitals with their own clinical laboratories. In South Korea, the test utilization committee of the Korean Society for Laboratory Medicine developed and published guidelines for test utilization in 2020 that mainly focus on testing under hospital conditions.^8^ Example indicators for the management of test utilization in these Korean guidelines usually focus on clinical laboratories in hospital settings.^8^ These findings suggest that rates of test utilization may be different between university hospitals and local clinics and further effective communication incorporating current authorized guidelines with physician knowledge are needed to facilitate more appropriate adoption of assays and to improve patient health care.

Although the exact number of cases of macrocytic anemia was not available, using the Korean Classification of Disease code on the Healthcare Bigdata Hub developed by the Health Insurance Review and Assessment Service in South Korea, about 5,000 patients/year with vitamin B12 deficiency anemia (D51 per the International Statistical Classification of Diseases and Related Health Problems, 10th revision code), about 3,500 patients/year with folate deficiency anemia (D52), and about 5,500 patients/year with other nutritional anemia (D53) were cared for from 2017 to 2019. When reviewing results pertaining the D531 code for other megaloblastic anemia (not otherwise specified), more than 1,700 patients/year visited clinics and hospitals from 2017 to 2019. Considering the underutilization of vitamin B12, folate, homocysteine, and methylmalonic acid assays in association with vitamin B12 and folate deficiency in adult patients with macrocytic anemia in local clinics and hospitals identified in this study, the disease burden of vitamin B12 and folate deficiency may be altered when tests became more widely adopted.^9^

The limitation of this study is that certain clinical data including a detailed history, physical examination, and other laboratory and image studies associated with macrocytic anemia or vitamin B12 or folate deficiency were not gathered. However, this study provides valuable information about rates of test utilization in local clinics and hospitals without in‐house clinical laboratories in South Korea. Further, the results of this study offer a basic overview regarding conducting assays in association with vitamin B12 and folate deficiency to improve test utilization in patients with macrocytic anemia in local clinics and hospitals without in‐house clinical laboratories in Korea. Considering that the most significant clinical effect of properly performed laboratory tests is improved patient outcomes, future studies are needed to clarify the clinical impact of appropriate test utilization of vitamin deficiency assays.^6^

## CONFLICTS OF INTEREST

The authors declare no conflict of interest.

## AUTHOR CONTRIBUTIONS

All authors contributed to article preparation. Rihwa Choi contributed to conceptualization; Rihwa Choi contributed to data curation; Rihwa Choi contributed to formal analysis; Sang Gon Lee and Eun Hee Lee contributed to funding acquisition; Rihwa Choi, Youngju Oh, Mi‐Jung Park, Youngrae Kim, and Serim Kim contributed to investigation; Rihwa Choi, Youngju Oh, Mi‐Jung Park, Youngrae Kim, and Serim Kim contributed to methodology; Sang Gon Lee contributed to resources; Sang Gon Lee and Eun Hee Lee contributed to supervision; Rihwa Choi contributed to validation; Rihwa Choi contributed to writing—original draft; Rihwa Choi, Sang Gon Lee, and Eun Hee Lee contributed to writing—review and editing. All authors read and approved the final article.

## Data Availability

The datasets generated and analyzed during the current study are available from the corresponding authors on reasonable request.
